# High stringency evaluation of the inactivation / exclusion efficacy of a MALDI-TOF MS chemical extraction method, with filtration of extract through 0.1 µm filters, on *Bacillus anthracis* Vollum vegetative cells and spores

**DOI:** 10.1371/journal.pone.0177294

**Published:** 2017-05-08

**Authors:** Simon A. Weller, Jennie Latham

**Affiliations:** CBR Division, Defence and Science Technology Laboratory, Ministry of Defence, Porton Down, Salisbury, United Kingdom; Centre National de la Recherche Scientifique, FRANCE

## Abstract

A previous report indicated that a formic acid chemical extraction method for the preparation of protein extracts for matrix-assisted laser desorption time-of-flight mass spectrometry (MALDI-TOF MS) identification, with filtration of extracts through 0.2 μm regenerated cellulose (RC) filters, would not reliably inactivate or exclude *Bacillus anthracis* Vollum cells or spores when tested under high stringency conditions. *B*. *anthracis* was recovered from 13/36 extracts (3/18 from vegetative cell extracts and 10/18 from bacterial spore extracts). In this paper we report the repetition of this study but with the substitution of the 0.2 μm, regenerated cellulose, filters with 0.1 μm polyvinylidene fluoride (PVDF) filters. Experiments were conducted under the same high stringency post-treatment viability test methods (100% of resulting protein content; 7 days Luria (L)-broth and a further 7 days L-agar plate incubation; or 7 days L-agar plate only incubation). *B*. *anthracis* was not recovered from any of 18 replicates generated from high concentrations of vegetative cells (10^7^ to 10^8^ cfu), but a single *B*. *anthracis* colony was recovered from one of 18 replicates generated from high concentrations of bacterial spores (10^8^ cfu), using a post-treatment viability culture method of 7 days on L-agar plate only. We discuss our results in the context of other similar studies and also a requirement to develop standardised post-treatment viability test methods.

## Introduction

Matrix-assisted laser desorption / ionisation time-of-flight mass spectrometry (MALDI-TOF MS) is used in microbiology for the identification of bacterial or fungal cultures [1]. The mass spectra produced from the protein content of isolates are reproducible, quickly generated, and libraries of spectra have been created from the analysis of known strains allowing the rapid identification of unknown isolates. Sample preparation can simply involve the transfer of isolated culture (e.g. with a toothpick) to the target plate and overlaying with HCCA matrix (α-Cyano-4-hydroxycinnamic acid)—a direct sample application. Chemical extraction methods can also be performed and have been shown to increase the number of successful identifications, especially for Gram-positive bacteria [2]. Typically 1 μL volumes of chemical extract are dried onto a target plate, overlain with 1 μL matrix, and tested.

When analysing cultures which potentially contain Highly Pathogenic Bacteria [HPB] (i.e. Advisory Committee on Dangerous Pathogens [ACDP] Hazard Group [HG] 3 [or Risk Group 3] agents requiring ACDP Containment Level [CL] 3 / Biosafety Level (BSL) -3 containment), consideration must be given to the inactivation of the culture being tested prior to MALDI-TOF MS identification. This is owing to the size of MALDI-TOF systems and required manufacturer engineering support making it generally impractical to operate a mass spectrometer within BSL-3 containment. Therefore, complete inactivation, or the ‘rendering safe’, of HPB in extracts prior to MALDI-TOF MS identification is the only reasonable alternative. Various studies have examined the bacterial inactivation efficacy of MALDI-TOF MS chemical extraction methods [3], with significant differences in method, post-treatment viability tests, and end results apparent between studies.

In a previous study [4] the inactivation efficacy of a MALDI-TOF chemical extraction method (using ethanol, formic acid, acetonitrile, and filtration), on high concentrations (up to 10^8^ colony forming units [cfu], per extract) of *Bacillus anthracis* vegetative cells and spores was examined. This method included double filtration of the resulting chemical extract through fresh 0.2 μm bacteriological filters, whose filter membranes was constructed of regenerated cellulose (RC). The inactivation efficacy was assessed with high stringency post-viability tests (100% of resulting protein content; multiple replicates; 7 day broth then 7 day agar plate culture; 7 day plate culture only). *B*. *anthracis* was recovered in 3/18 vegetative cell extracts and 10/18 spore extracts indicating the method could not be relied upon to exclude pathogenic *B*. *anthracis* from extracts.

In this paper we report the repetition of the previous study but with the substitution of the 0.2 μm, regenerated cellulose, filters with 0.1 μm bacteriological filters, whose filter membranes were constructed of polyvinylidene fluoride (PVDF). Experiments were conducted under the same high stringency post-treatment viability test methods to determine if the decreased filter pore size and different membrane material would result in an increase in the inactivation efficacy, or exclusion, of *B*. *anthracis* from extracts.

## Materials and methods

Experimental procedures followed those conducted in the previous study [4] with the substitution of 0.2 μm filters with 0.1 μm filters. The use of the virulent (pXO1^+^; pXO2^+^) *B*. *anthracis* Vollum strain was approved by the Dstl Microbiological Safety Technical Authority. Under ACDP CL3 conditions vegetative cells were cultivated overnight (37°C) on L-agar plates. None of the cultures used were more than 24 hours old to help prevent sporulation. Vegetative cell suspensions were enumerated by the production of a 10-fold dilution series and plating of appropriate dilutions (100 μL aliquots; 3 reps per dilution) onto L-agar plates. These plates were incubated (min. 48 hours) and colonies counted. Spore extracts were prepared on New Sporulation Medium from a pre-existing, enumerated, suspension of *B*. *anthracis* Vollum spores. These extracts were produced on New Sporulation medium (NSM), and washed and stored (at -20°C) in distilled water using a previously reported method [5].

Plate cultures were re-suspended into 1 mL of sterile 1 × Dulbeccos Phosphate Buffered Saline (1 x DPBS). MALDI extracts were then prepared using the formic acid and acetonitrile method as found in the MALDI-TOF MS Biotyper system (Bruker) user manual [6]. One hundred μL aliquots of each suspension were added to 900 μL of 70% Ethanol. The suspension was mixed thoroughly, centrifuged (13 000 rpm; 2 min; Eppendorf MiniSpin Plus) and the supernatant discarded. The pellet was then re-suspended in 50 μL of 70% formic acid and mixed thoroughly. Fifty μL of 100% acetonitrile was then added, the suspension mixed thoroughly, and centrifuged (13 000 rpm; 2 min). The supernatant was then passed (7 000 rpm; 10 secs) through a 0.1 μm Ultrafree® MC VV, PVDF membrane, centrifugal spin column (Merck Millipore Ltd, Cork, Ireland). The resulting filtrate was then passed (7 000 rpm; 10 secs) through a fresh 0.1 μm Ultrafree® spin column and the chemical extract was retained for post treatment viability testing. For spore extracts 100 μL aliquots were processed in the same way.

Entire protein filtrates were pipetted into the wells of a sterile 6-well tissue culture plate (Corning Costar), and spread around the base of each well with a sterile cell scraper. Extracts were then allowed to dry (typically for 20–30 mins) to ensure evaporation of the formic acid and acetonitrile and prevent carry over into culture. Resulting protein extract was then re-suspended by adding 100 μL aliquots of sterile 1 × DPBST (DPBS with 0.01% Tween) to each well. The aliquot was applied across the well by repeated pipetting (30 secs) to ensure coverage of the entire well base and re-suspension of as much protein extract as possible. All 1 × DPBST was then removed from each well and put into culture (see below). Positive controls comprised cell suspensions prepared following the above method—until the filtration steps–but with substitution of all reagents with 1 × DPBS. The final bacterial pellet (in 1 x DPBS) was re-suspended and added to a well, as before, and allowed to dry. A negative control (addition of 100 μL of sterile 1 × DPBST to a naïve well) was also prepared and cultured in each experiment. Each experiment comprised 3 MALDI-TOF protein extracts, one positive control, and one negative control. In total eighteen MALDI-TOF extract replicates were produced from each cell type, with two different methods of post-treatment viability testing undertaken.

One method of post-treatment viability testing was designed to provide a qualitative indication (0.1 μm QUAL experiments) of the inactivation efficacy of the method. Nine protein extract replicates from each cell type (re-suspended into 1 × DPBST) were added to 900 μL of L-broth and incubated (7 days; 37°C). Entire broths were then plated onto L-agar (250 μL × 4 plates) and incubated (a further 7 days; 37°C). The other post-treatment viability test was designed to provide a quantitative indication (0.1 μm QUANT experiments) of the inactivation efficacy of the treatment. Nine replicates of each cell type protein extract (re-suspended into 1 × DPBST) were directly plated onto L-agar plates and incubated (7 days; 37°C).

All final plates were witnessed by a Dstl staff member not directly involved in the study. Any resulting colonies were re-isolated onto fresh L-agar plates and incubated (37°C). A 1 μL loop of each resulting culture was added to 1 mL sterile distilled water and heated (99°C; 15 mins). One μL aliquots of the resulting boilates were tested by the chromosomal (BA chr-MGB) and pXO1 (pXO1-MGB) targeted PCR assays [7], with a *B*. *anthracis* Vollum DNA extract used as a positive control.

## Results

Results from vegetative cell experiments are summarised in Table 1. *B*. *anthracis* was not recovered from any of the 18 replicates. Results from spore experiments are summarised in Table 2. A single *B*. *anthracis* colony was recovered from 1 of the 18 replicates. This was from a quantification experiment (0.1 μm QUANT MALDI S1; Extract 2) where the extracts were not incubated in L-broth. No *B*. *anthracis* colonies were recovered from any of the other experiments (0.1 μm QUAL or QUANT). The re-isolate from 0.1 μm QUANT MALDI S1, Extract 2) tested positive for *B*. *anthracis* chromosomal and pXO1 real-time PCR targets using previously published assays [7].

**Table 1:** Summary of *B*. *anthracis* recovery, from dried MALDI protein extracts derived from vegetative cells, in 0.1 μm QUAL and 0.1 μm QUANT experiments.

Experiment and *B*. *anthracis* input amount	Extract or control	*B*. *anthracis* L-agar plate colony counts / coverage estimate^a^			
		Plate 1	Plate 2	Plate 3	Plate 4
0.1 μm QUAL MALDI 1	Extract 1	0	0	0	0
2.03 × 10^8^ cfu	Extract 2	0	0	0	0
	Extract 3	0	0	0	0
	Positive	TMTC (>95%)	TMTC (>95%)	TMTC (>95%)	TMTC (>95%)
	Negative	0	0	0	0
0.1 μm QUAL MALDI 2	Extract 1	0	0	0	0
2.33 × 10^8^ cfu	Extract 2	0	0	0	0
	Extract 3	0	0	0	0
	Positive	TMTC (>95%)	TMTC (>95%)	TMTC (>95%)	TMTC (>95%)
	Negative	0	0	0	0
0.1 μm QUAL MALDI 3	Extract 1	0	0	0	0
2.0 × 10^8^ cfu	Extract 2	0	0	0	0
	Extract 3	0	0	0	0
	Positive	TMTC (>95%)	TMTC (>95%)	TMTC (>95%)	TMTC (>95%)
	Negative	0	0	0	0
0.1 μm QUANT MALDI 1	Extract 1	0	n/a^b^	n/a	n/a
5.47 × 10^7^ cfu	Extract 2	0	n/a	n/a	n/a
	Extract 3	0	n/a	n/a	n/a
	Positive	TMTC (>95%)	n/a	n/a	n/a
	Negative	0	n/a	n/a	n/a
0.1 μm QUANT MALDI 2	Extract 1	0	n/a	n/a	n/a
2.33 × 10^8^ cfu	Extract 2	0	n/a	n/a	n/a
	Extract 3	0	n/a	n/a	n/a
	Positive	TMTC (>95%)	n/a	n/a	n/a
	Negative	0	n/a	n/a	n/a
0.1 μm QUANT MALDI 3	Extract 1	0	n/a	n/a	n/a
1.03 × 10^8^ cfu	Extract 2	0	n/a	n/a	n/a
	Extract 3	0	n/a	n/a	n/a
	Positive	TMTC (>95%)	n/a	n/a	n/a
	Negative	0	n/a	n/a	n/a

^**a**^ TMTC = too many to count; Coverage estimate (in parentheses): basic estimate of total coverage of plate by bacterial colonies or lawn.

^b^ n/a = not applicable. Only one L-agar plate, per rep, was inoculated in QUANT experiments.

**Table 2:** Summary of *B*. *anthracis* recovery, from dried MALDI protein extracts derived from spores, in 0.1 μm QUAL and 0.1 μm QUANT experiments.

Experiment and *B*. *anthracis* input amount	Extract or control	*B*. *anthracis* L-agar plate colony counts / coverage estimate^a^			
		Plate 1	Plate 2	Plate 3	Plate 4
0.1 μm QUAL MALDI S1	Extract 1	0	0	0	0
5.4 × 10^8^ cfu	Extract 2	0	0	0	0
	Extract 3	0	0	0	0
	Positive	TMTC (>95%)	TMTC (>95%)	TMTC (>95%)	TMTC (>95%)
	Negative	0	0	0	0
0.1 μm QUAL MALDI S2	Extract 1	0	0	0	0
5.4 × 10^8^ cfu	Extract 2	0	0	0	0
	Extract 3	0	0	0	0
	Positive	TMTC (>95%)	TMTC (>95%)	TMTC (>95%)	TMTC (>95%)
	Negative	0	0	0	0
0.1 μm QUAL MALDI S3	Extract 1	0	0	0	0
5.4 × 10^8^ cfu	Extract 2	0	0	0	0
	Extract 3	0	0	0	0
	Positive	TMTC (>95%)	TMTC (>95%)	TMTC (>95%)	TMTC (>95%)
	Negative	0	0	0	0
0.1 μm QUANT MALDI S1	Extract 1	0	n/a^c^	n/a	n/a
5.4 × 10^8^ cfu	Extract 2	1^b^	n/a	n/a	n/a
	Extract 3	0	n/a	n/a	n/a
	Positive	TMTC (>95%)	n/a	n/a	n/a
	Negative	0	n/a	n/a	n/a
0.1 μm QUANT MALDI S2	Extract 1	0	n/a	n/a	n/a
5.4 × 10^8^ cfu	Extract 2	0	n/a	n/a	n/a
	Extract 3	0	n/a	n/a	n/a
	Positive	TMTC (>95%)	n/a	n/a	n/a
	Negative	0	n/a	n/a	n/a
0.1 μm QUANT MALDI S3	Extract 1	0	n/a	n/a	n/a
2.7 × 10^8^ cfu	Extract 2	0	n/a	n/a	n/a
	Extract 3	0	n/a	n/a	n/a
	Positive	TMTC (>95%)	n/a	n/a	n/a
	Negative	0	n/a	n/a	n/a

^**a**^ TMTC = too many to count; Coverage estimate (in parentheses): basic estimate of total coverage of plate by bacterial colonies or lawn.

^b^ Confirmed as *B*. *anthracis* by Ba chr and pXO1-MGB PCRs [7].

^c^ n/a = not applicable. Only one L-agar plate, per rep, was inoculated in QUANT experiments.

## Discussion

As in the previous study *B*. *anthracis* Vollum was chosen for the high stringency inactivation experiments in order to provide consistency and because the bacterial spore is considered to be one of the hardest organism types to inactivate [8]. Under institutional guidelines and defined stringent post-treatment viability test protocols, *B*. *anthracis* (in both cell and spore form) was also used as a model agent to generate high confidence in the method; if total inactivation or exclusion across multiple replicates was observed. The method could then be used against subsequent samples without the requirement for further sterility checks on individual extracts, regardless of the bacterial species being tested.

When compared to the results of the previous study (summarised in Table 3) the substitution of 0.2 μm filters with 0.1 μm filters provided a clear reduction in the number of replicates from which *B*. *anthracis* was recovered (1/36 extracts [0.1 μm filters] from 13/36 extracts [0.2 μm]), despite similar bacterial loads being treated in both studies (Spore experiments: 5.4–2.7 × 10^8^ cfu [0.1 μm filters] vs. 1.2 × 10^8^ cfu [0.2 μm filters]; Vegetative cell experiments: 5.47 × 107–2.03 × 10^8^ cfu [0.1 μm] vs. 6.7 × 106–6.7 × 10^8^ cfu [0.2 μm]). In addition, unlike the 0.2 μm filter study, no recovery was observed from extracts prepared from vegetative cells. This is especially significant in the context of clinical laboratories where MALDI-TOF is used to identify fresh cultures (i.e. from positive blood culture bottles), and therefore where testing of high concentrations of bacterial spores is unlikely.

**Table 3:** Summary of results from 0.1 μm (2016) and 0.2 μm (2015) filter experiments.

Experiment Type	Cell Type	Recovery of *Bacillus anthracis* from experimental replicates^a^	
		0.1 μm filters^b^	0.2 μm filters^c^
QUAL MALDI	Vegetative cells	0/9	3/9
QUANT MALDI	Vegetative cells	0/9	0/9
QUAL MALDI S	Spores	0/9	6/9
QUANT MALDI S	Spores	1/9	4/9

^a^ Number of replicates where *B*. *anthracis* was recovered (and quantity)

^b^ Results from this study

^c^ Results from previous study [4]

This was a purely observational study and whilst there are several possible explanations for the recovery of a single *B*. *anthracis* colony from a spore sample extract, the actual functional mechanism of this escape is unknown. Although *B*. *anthracis* spores have been measured with mean diameters 0.81–0.86 μm and mean lengths of 1.26–1.67 μm [9], and therefore should not pass through a 0.1 μm filter membrane, another study observed a 1% failure rate when filtering *B*. *anthracis* spore suspensions (10^6^ cfu in PBS) through a 0.1 μm Ultrafree MC filter [10]. This failure rate was only eliminated when centrifugal forces were reduced below 10000 ×*g*. However, in our study, (with centrifugation at 7 000 rpm and rotor radius of 6 cm), we calculated that the Ultrafree filters were centrifuged at 3287 ×*g* (for 10 second intervals) using a published conversion table [11]. The filter membrane material used in this study, PVDF, is recommended for use with acetonitrile by the manufacturer of the UltraFree filters [12] and although no mention is made of compatibility with formic acid in this guide, other manufacturers of PVDF do state resistance to this chemical [13]. With extracts also having being passed through two filtration steps (with fresh filters being used each time), then it is unclear as to what attribute of the filters themselves could have allowed passage of a *B*. *anthracis* spore.

Of other possible escape mechanisms action of formic acid and acetonitrile on spore structure is possible, modifying the spore to allow passage through the membrane. However, we have not conducted a microscopic evaluation of the effect of these chemicals on the spore structure. We also think it unlikely that experimental error could account for the single recovery as negative controls have always remained clear (24 negative controls across both 0.1 μm and 0.2 μm studies) and contamination from the high concentration positive controls would likely result in the recovery of more than a single colony. This indicates that the experimental methodology is robust. Therefore in summary, we have not identified the mechanism which has allowed the recovery of this single colony from the 36 experimental replicates generated in this study. As noted in a recent review [3] there has been no systematic study on the effect of chemical action on bacterial filters or the bacterial spore / cell in MALDI-TOF MS extracts which may lead to escape of viable cells during the extraction process. This study has not addressed this.

As also noted in the recent review [3] different studies on the inactivation efficacy of MALDI-TOF chemical extraction methods have employed varying post-treatment viability tests. In the previous [0.2 μm filter] study *B*. *anthracis* was recovered from more extracts which were tested with a broth and agar plate culture viability test method (9/18 extracts), than those which had only undergone agar plate culture (4/18 extracts), supporting previous reports which had suggested that a broth culture stage can enhance the recovery of treated, sub-lethally injured, *Bacillus* cells [14, 15], and which is now incorporated into guidance provided by the US Federal Select Agent Program [16]. In the current study the only recovered strain was from a sample that had only undergone the 7 day agar plate culture method. Whether this means that a broth recovery stage is not required for a stringent post-treatment viability test of *B*. *anthracis* samples is unclear. However, as stated in the previous review [3], and in other reports [17], the development of optimal, standardised, protocols for post-treatment viability tests for the recovery of bacterial species, whether this be *B*. *anthracis* (if used as a model agent to inform the applicability of a method to all bacterial species) or another bacterial species is required. A recent study also tested the inactivation efficacy and MALDI-TOF MS compatibility of the formic acid extraction method against a panel of HPB [18], with extracts also being filtered (though only once) through the Millipore MC 0.1 μm, PVDF, filters. In this study all 31 extracts were shown to be free of viable agent, though post treatment viability tests methods were different than our study with 10% of each extract being tested and different culture conditions; 2 day agar plate only or 2 day broth and 3 day agar plate depending on agent. Therefore systematic studies which answer questions such as i) can the results from a model agent (such as *B*. *anthracis*) be transferred to all other agents?; ii) can a method, and therefore 100% of the sample, be tested rather than sterility checking individual samples?; iii) is a broth culture recovery stage necessary?, iv) what is an optimal culture incubation period?; could lead to the development of standardised post viability test methods which allow direct comparison of results across studies.

A remaining question from our study regards the significance of recovering a single colony from one of 36 extracts—all generated from bacterial suspensions in the range of 10^7^−10^8^ cfu, and none from fresh vegetative cells. The starting volume of the extracts (prior to filtration) was a 100 μL supernatant. MALDI-TOF MS requires fresh culture and 1 μL volumes of chemical extract to test. Therefore it might be said that an extract prepared under CL3 conditions could be removed, as a small volume aliquot, and considered to have been ‘rendered safe’ with a resulting minimal risk of infection to an operative.

The results generated in this study will inform the development of institutional protocols to allow inactivation and sample processing of HG3 agents (in a CL3 laboratory), with subsequent analysis using a MALDI-TOF MS system in a CL2 laboratory. To achieve this a model agent was used to inform the applicability of a method to all bacterial species. Application of this method in other institutes would be at the discretion of individual safety committees, as would the use of model agent. Although further information on the utility of the method would be gained by testing against alternate bacterial species, systematic testing of the method (with equivalent post-treatment viability test methods) in other institutes would also help identify any weak points from which escape of treated bacterial agents is possible / likely. These could be factors associated with consumables / reagents, or points in the process likely to be susceptible to operative error.

In conclusion, this study will contribute to a recent body of research, review, and incident investigation [3, 4, 17, 18, 19] into the inactivation of MALDI-TOF MS extracts and could aid both the development of standardised post-treatment viability tests and species agnostic extraction methods. For example the manual [3] for the Biotyper MALDI-TOF MS system (Bruker) suggests different extraction methods (though without defining the requirement for filtration that has been identified in this and other studies) should be used for pathogenic and spore-forming agents. This indicates that prior knowledge of the agent before testing is required and potentially reduces the power of the technology to safely identify an unknown culture. A universal method that could safely generate a chemical extract from all bacterial strains could therefore be of benefit to a range of clinical and diagnostic laboratories. Whether this method be the formic acid method or another method such as a triflouroacetic acid TFA protocol [20], it could be applied with confidence, and perhaps be automated, to further minimise any risk of exposure to the operative.
